# Comparation of robotic-assisted surgery and laparoscopic‑assisted surgery in children with Hirschsprung’s disease: a single-centered retrospective study

**DOI:** 10.1186/s12893-023-02169-2

**Published:** 2023-09-26

**Authors:** Shuhao Zhang, Duote Cai, Yuebin Zhang, Tao Pan, Ken Chen, Yi Jin, Wenjuan Luo, Zongwei Huang, Di Hu, Qingjiang Chen, Zhigang Gao

**Affiliations:** Department of General Surgery, The Children’s Hospital, Zhejiang University School of Medicine, National Clinical Research Center for Child Health, Hangzhou, 310003 China

**Keywords:** Hirschsprung’s disease, Robotic-assisted surgery, Laparoscopic-assisted surgery, Enterocolitis

## Abstract

**Background:**

There are few studies comparing robotic-assisted surgery (RAS) and laparoscopic-assisted surgery (LAS) in Hirschsprung’s disease (HSCR). This study aimed to compare intraoperative and postoperative outcomes between RAS and LAS performed during the same period.

**Methods:**

All consecutive 75 patients with pathologically diagnosed as HSCR who underwent Swenson pull-through surgery from April 2020 to Nov 2022, were included. Patients were divided into RAS group and LAS group and a retrospective analysis was performed based on clinical indexes and prognosis.

**Results:**

A total of 75 patients were included, among which, 31 patients received RAS and 44 received LAS. The RAS and LAS groups had similar ages, sex, weight, postoperative hospital stays, and fasting times. Compared with LAS, blood loss (*p* = 0.002) and the incidence of Hirschsprung-associated enterocolitis (*p* = 0.046) were significantly lower in the RAS group. The first onset of Hirschsprung-associated enterocolitis in patients younger than 3 months occurred significantly earlier (*p* = 0.043). Two patients experienced anastomotic leakage in the LAS group and one patient experienced incisional hernia in the RAS group. The cost of RAS was significantly higher than that of LAS (*p* < 0.0001).

**Conclusions:**

RAS is a safe and effective alternative for HSCR children, and a delaying primary surgery until later in infancy (> 3 months) may improve outcomes.

## Introduction

Hirschsprung disease (HSCR) is a rare congenital anomaly of the enteric nervous system, characterized by the absence of ganglionic cells in the distal bowel. Since the first successful treatment of HSCR was reported in 1948 [1], the surgical approach to radical surgery has changed from laparotomy surgery (LS) to mini-invasive surgery in almost all the pediatric centers.

The first laparoscopic colon pull-through was performed by Soave and Duhamel in 1994 [2, 3]. Since then, laparoscopic techniques have rapidly developed worldwide. Compared with LS, laparoscopic-assisted surgery (LAS) has the advantages of reduced pain, improved cosmesis, and a shorter length of hospital stays. In 2001, robotic surgery was first reported in children and in 2011, Hebra et al. [4]. provided the first report of Robotic Swenson pull-through for HSCR in infants. Recently, related studies of robotic-assisted treatments of HSCR have been reported. However, to date, there are few studies comparing robotic-assisted surgery (RAS) and LAS for the treatment of HSCR. This study, therefore, set out to assess the differences in intraoperative and postoperative outcomes between RAS and LAS in treating HSCR.

## Materials and methods

### Study design

Data from a total of 75 HSCR patients who were treated at the Department of General Surgery, Children’s Hospital of Zhejiang University School of Medicine from April 2020 to November 2021 were collected. All operations were performed by our experienced surgeons. Variables including sex, age, weight, enterostomy, operative time, classification of HSCR, blood loss, hospital stays, use of an abdominal drainage tube, cost, and postoperative outcomes were recorded. A comparative analysis was performed between the RAS group and LAS group based on the above clinical data and prognosis.

### Inclusion criteria

The inclusion criteria included: 1) Patients were diagnosed with HSCR based on rectal biopsy, intraoperative fast frozen section, and postoperative pathological results; 2) the Children’s Hospital of Zhejiang University School of Medicine was the first hospital that patients visited; and 3) patients did not have a history of any other abdominal surgeries.

### Definition of HAEC (Hirschsprung-associated enterocolitis)

The classic features of HAEC include abdominal distention, fever, and diarrhea. However, other non-specific symptoms including vomiting, rectal bleeding, lethargy, loose stools, and obstipation also may be the indication of HAEC. Here, we use the diagnosis and grading scale proposed by Gosain et al. [5] as our criteria of HAEC. All patients diagnosed with HAEC (Grade II-III) were hospitalized.

### Surgical technique and docking position

**Fig. 1:**
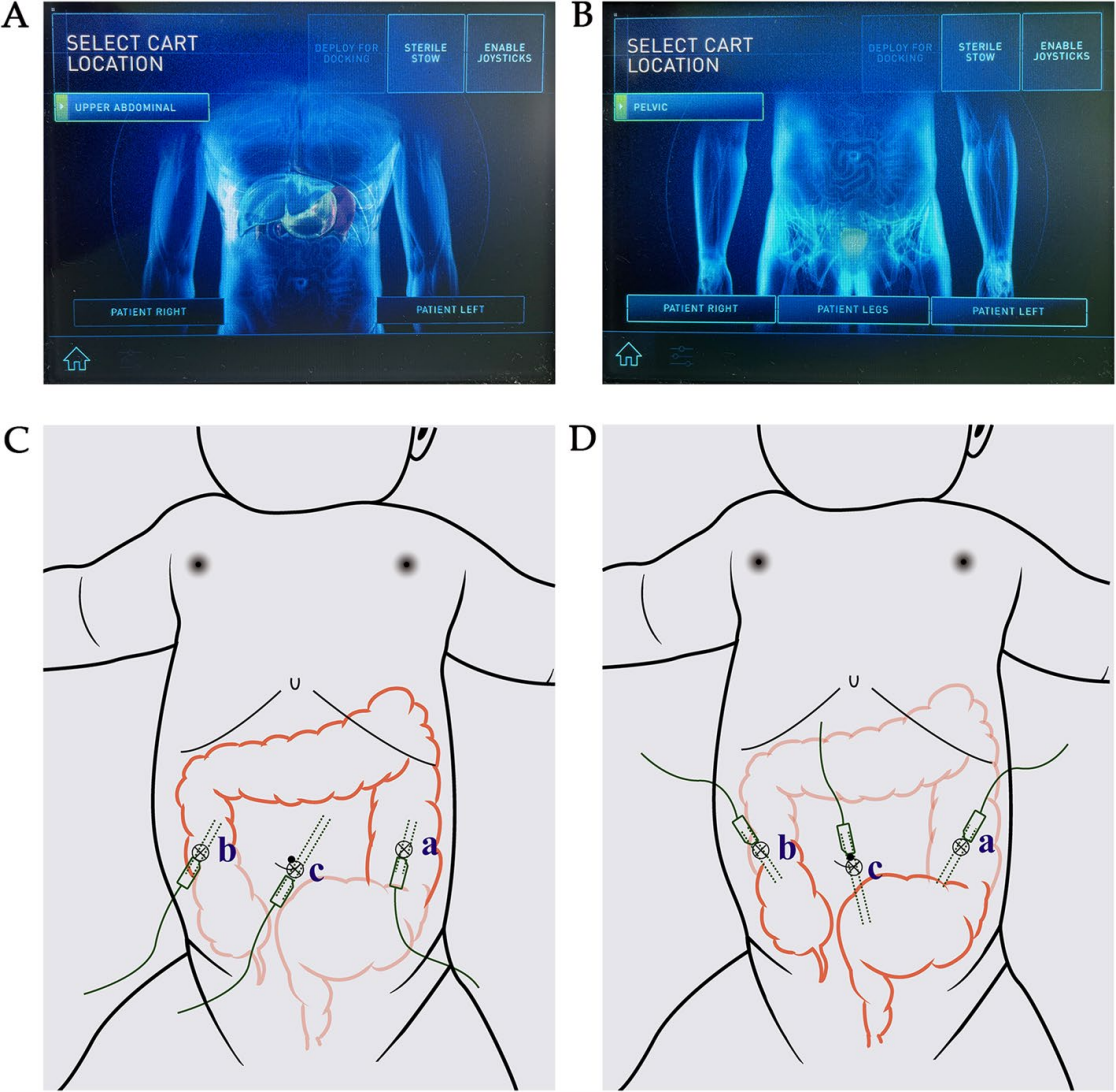
Robotic programs of different surgical positions and port placements. **A** and **C**: Program of Upper abdominal location. **B** and **D**: Program of pelvic location. c is camera port (12 mm), a and b are working ports (8 mm)

The DaVinci Xi robotic system was used for 31 procedures performed at the Children’s Hospital of Zhejiang University School of Medicine. When dealing with long-segment or total-colonic HSCR, the pelvic position (Fig. 1D) was first set to release the intestine from the pelvic floor to the splenic flexure of the colon, and then from the ileocecal portion to the hepatic flexure of the colon. Then, we reinstalled the robotic system to the epigastric position (Fig. 1C) and released the intestine from the splenic flexure to the hepatic flexure of the colon. The docking positions are shown in Fig. 1.

### Statistical analysis

Frequencies and percentages were used to describe categorical variables; continuous variables were presented as the median (interquartile range). Significant differences between two groups were tested by the Pearson’s Chi-square test for categorical variables, and the Mann–Whitney U test for continuous variables. All the data and analyses were performed by SPSS 22.0 and Graphpad Prism 6, and statistical significance was defined as *p* < 0.05.

## Results

The two groups of patients were similar in age, sex, and weight. The total average operative time of RAS and LAS was not significantly different, however, the median time of RAS was shorter than that of LAS (125.0 min (111.0–143.0) vs. 135.0 min (106.8–160.0)), while the median time of RAS in dealing with Classic HSCR and short segment HSCR was longer than that of LAS (124.0 min (111.0–139.8) vs. 110.5 min (101.8–155.8)). Within the RAS group, there are 28 cases of C-HSCR patients and 3 cases of L-HSCR patients. Within the LAS group, there are 24 cases of C-HSCR patients, 16 cases of L-HSCR patients, and 4 cases of S-HSCR patients (Table 1).

**Table 1:** Demographics of patients who underwent robotic surgery or laparoscopic surgery

	RAS	LAS	*p* value (LAS vs. RAS)
Sex (F: M)	6: 25	9: 35	R^2^ = 0.014 *p* = 0.907
Age (month)	4.8 (2.3–22.0)	3.4 (2.2–21.5)	0.4748
Weight (kg)	7.2 (5.1–10.5)	6.4 (5.0–11.1)	0.5328
Enterostomy			/
Intestinal enterostomy	/	1	
Colostomy	/	2	
Operative time (min)			
Total	125.0 (111.0–143.0)	135.0 (106.8–160.0)	0.5128
L-HSCR	171.0	150 (141.3–226.8)	/
C-HSCR, S-HSCR	124.0 (111.0–139.8)	110.5 (101.8–155.8)	0.2439
Classification			/
C-HSCR	28	24	
S-HSCR	0	4	
L-HSCR	3	16	
T-HSCR	0	0	

*C-HSCR* Classic HSCR, *S-HSCR* Short-segment HSCR, *L-HSCR* Long-segment HSCR, *T-HSCR* Total colonic HSCR

**Fig. 2:**
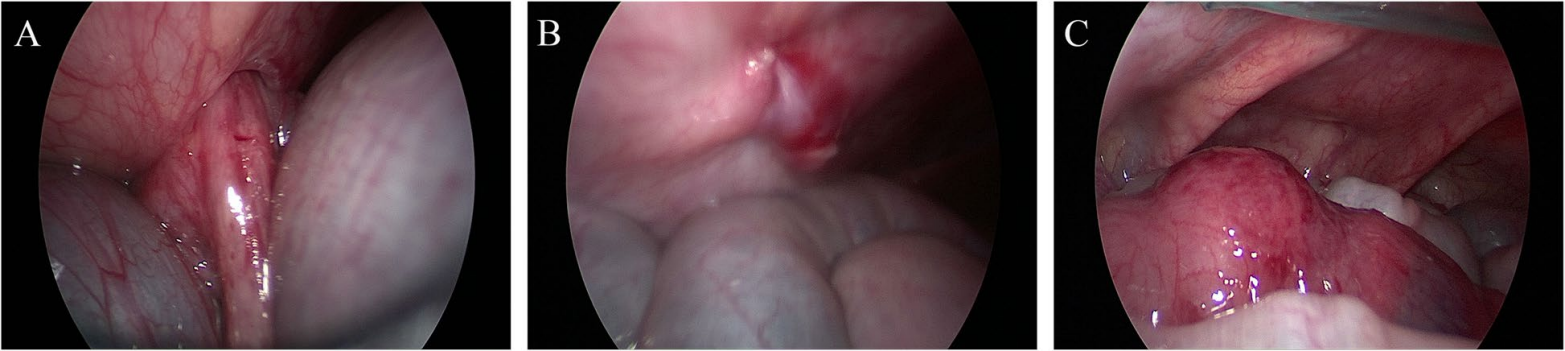
Intraoperative photographs of incisional hernia in RAS group. **A** The incarcerated intestine. **B** The re-sutured robotic surgical incision. **C** Necrosis was not seen in the incarcerated intestine

The postoperative hospital stays and fasting times were not significantly different between the RAS and LAS groups (*p* = 0.4466 and 0.9485, respectively). The RAS group experienced a lower mean estimated blood loss (3.0 ml (2.0–4.9) vs. 5.1 ml (4.8-0.9.0); *p* = 0.002) and higher hospitalization costs (10332.0 USD (9576.3–12208.7) vs. 4029.6 USD (3289.9–5527.3), *p* < 0.0001) compared with the LAS group. Two patients experienced postoperative anastomotic leakage in the LAS group. There were three patients underwent enterostomy in LAS group. After improving the patient's condition through nutritional support and cleaning up the depositional fecalith in the intestines, all the three patients received successful radical surgery without intraoperative complications. The postoperative follow-up time for the RAS group and LAS group was 17.7 (6.8–19.3) months and 20.8 (18.1–25.3) months, respectively. During the follow-up period, 17 patients in the LAS group experienced HAEC, while the RAS group had only 3 cases of HAEC, which was significantly lower than the LAS group (9.7% vs. 38.6%, *p* = 0.046) (Table 2). Although the incidence of HAEC between patients older (group A) or younger (group B) than three months of age was not significantly different (26.2% vs. 27.3%, *p* = 0.916), the first onset of HAEC in group B occurred significantly earlier than in group A (0.5 months (0.4–3.3) vs. 3.6 months (1.4–6.0), *p* = 0.043) (Table 3). Besides, two patients experienced postoperative anastomotic leakage in the LAS group and one patient experienced incisional hernia in the RAS group (Table 2 and Fig. 2).

**Table 2:** Outcomes of patients who underwent robotic surgery or laparoscopic surgery

	RAS	LAS	*p* value (LAS vs. RAS)
Blood loss (ml)	3.0 (2.0–4.9)	5.1 (4.8-.9.0)	0.002
Postoperative hospital stays (day)	7.5 (7.0–11.0)	7.5 (7.0–12.8)	0.4466
Fasting time (day)	3.0 (2.0–4.0)	2.0 (1.0–5.0)	0.9485
Cost (CNY)	75113.8.0 (69620.0–88757.3)	29295.8 (23917.8–40183.7)	*p* < 0.0001
Incidence of HAEC	9.7% (3/31)	38.6% (17/44)	R^2^ = 3.998 *p* = 0.046
Incidence of anastomotic leakage	0	4.5% (2/44)	/
Follow-up time (month)	17.7 (6.8–19.3)	20.8 (18.1–25.3)	/

*HAEC* Hirschsprung-associated enterocolitis

**Table 3:** Comparations of patients younger or older than three months

	> 3 months (group A)	< 3 months (group B)	*p* value
Sex (F: M)	7: 35	8: 25	R^2^ = 0.663 *p* = 0.416
Age (month)	19.5 (6.1–42.5)	2.2 (1.8–2.5)	/
Weight (kg)	10.3 (7.7–15.0)	5 (4.6–5.7)	/
Operative time (min)	130.0 (110.0–165.0)	115.0 (107.0–146.5)	0.1820
LAS: RAS	22:20	22:11	/
Classification			/
C-HSCR	30	22	
S-HSCR	4	0	
L-HSCR	8	11	
T-HSCR	0	0	
HAEC			
percentage	11/42 (26.2%)	9/33 (27.3%)	R^2^ = 0.011 *p* = 0.916
time of initial onset (month)	3.6 (1.4–6.0)	0.5 (0.4–3.3)	0.043

All patients will have outpatient follow-up at 2 weeks, 1 month, 2 months, 3 months, and 6 months after surgery. All patients who meet the criteria of Hirschsprung-associated enterocolitis (shown in Materials and Methods section) during the follow-up period will be admitted to the hospital for treatment. In the RAS group, one patient experienced abdominal pain 10 days after surgery. An outpatient ultrasound examination indicated an incisional hernia (incision b, seen in Fig. 1C), with echoes resembling intestine. The patient was admitted to the hospital for re-operation, and the surgical incision was re-sutured after the intestine was pushed back to the abdominal cavity (Fig. 2). Two patients with anastomotic leakage recovered after received conservative treatment including fasting, nutrition support, and anti-infective therapy (meropenem). The lengths of hospital stay of them were 26 days and 14 days, and the costs were 13,120.6 and 8,732.4 USD, respectively.

## Discussion

HSCR was first described by Harald Hirschsprung in older children in 1886. With the introduction of minimally-invasive surgery, classic techniques have been modified and improved by laparoscopic or robotic procedures with reduced pain, improved cosmesis, and shorter lengths of hospital stay. However, there is limited research comparing LAS and RAS which performed during the same period for the treatment of HSCR.

Since 1995, when Georgeson et al. first described LAS for HSCR in infants, many groups reported the benefits of this technique [6]. Laparoscopic-assisted endorectal colon pull-through using a variation of the Soave technique was adopted as the preferred procedure [7]. Compared with LAS, RAS overcomes many obstacles with its amplified 3-D visualization, 7-degree range of motion, tremor elimination and hand–eye coordination. In 2012, Hebra et al. [4]. reported 12 robotic-assisted Swenson procedures for the treatment of HSCR. Following that, Mattioli [8] and Prato [9] reported totally 14 Soave procedures for the treatment of HSCR in 2017 and 2020, respectively. The latest cohorts involving the robotic-assisted procedure were reported by Delgado-Miguel [10] and Quynh [11] in 2021, in which 67 patients underwent the robotic Soave pull-through procedure (Table 4). Among the collective 93 patients, 8 patients had HAEC and 2 patients had anastomotic stricture without any major intraoperative surgical issues or technical malfunctions. The total incidence (8/93, 8.6%) of HAEC was significantly lower than previously reported (from 25%-37%) [12, 13].

**Table 4:** Comparison of robotic-assisted surgery in Hirschsprung’s disease

	Number of operations	Operative time (min)	hospital stays (day)	HAEC (n)	Other complications (n)	Age (month)	Follow-up time (month)
Current study	31	125.0 (111.0–143.0)	7.5 (7.0–11.0)	3	Incisional hernia (1)	4.8 (2.3–22.0)	17.7 (6.8–19.3)
Delgado-Miguel [10]	15	240 ± 72	3 (3–4)	1	Anastomosis dehiscence (1) Constipation (2)	4 (3–6)	79 (45–115)
Quynh [11]	55	93.2 ± 35	5.5 (4–8)	4	Incontinence (3) Mild soiling (2)	24.5 (6–120)	43.2 (30–66)

*HAEC* Hirschsprung-associated enterocolitis

**Fig. 3:**
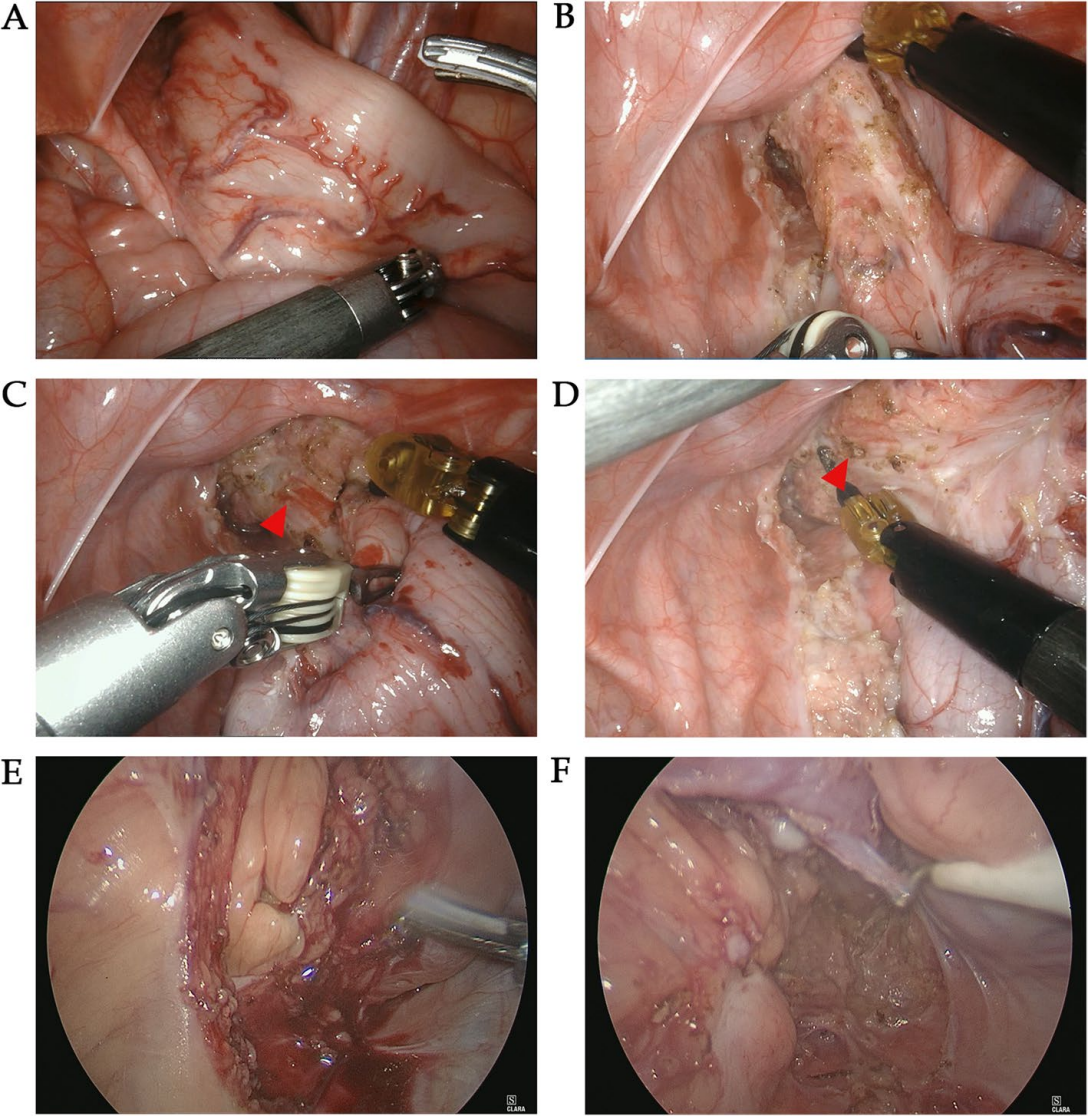
Intraoperative photographs of RAS and LAS. **A** and **B** The amplified and high-definition visualizations of pelvic floor and peritoneal reflection in RAS. **C** and **D** The accurate monopolar bleeding control in RAS. Red arrows point to the bleeding point. **E** and **F** Intraoperative photographs of LAS showed relatively unclear surgical field and poor identification of seromuscular

In our study, we compared RAS and LAS performed at the same period. As for the postoperative outcomes, RAS resulted in lower estimated blood loss. The results were likely to be due to the fact that the robotic system had tremor elimination and motion scaling functions, resulting in higher dexterity and stability. Thus, such factors could translate to lower accessory injury and injury-related exudation. Another important finding in this study was that we confirmed that the incidence of HAEC in RAS was significantly lower than that in LAS. As the leading cause of serious morbidity and death in HSCR patients, the exact etiology is still unknown, several hypotheses have been proposed including dysbiosis of the intestinal microbiome [14–16], impaired mucosal barrier function [17], altered innate immune responses [18], and bacterial translocation [19]. L-HSCR is an independent risk factor for the development of postoperative HAEC [20], and the significantly higher number of L-HSCR cases in the LAS group compared to the RAS group may also be one of the reasons for the higher incidence of HAEC in the LAS group. However, it is undeniable that the robotic system can help achieve an ideal identification and dissection of the seromuscular layer, vas deferens, ureters, and pelvic splanchnic nerve. Based on the clear anatomical plane, we performed accurate seromuscular dissection and preserved as many vessels and nerves of pelvic floor as possible, which might be one of the reasons for the decreased incidence of HSCR. Such advantages could be amplified in the limited working space deep in the pelvis and under the peritoneal reflection (Fig. 3). Especially in older children with recurrent preoperative HSCR, RAS could improve the identification of the vessels and nerves when dealing with pelvic adhesion. Early anastomotic leakage was also not encountered in our robotic series, while 4.5% of the LAS patients experienced anastomotic fistula. The robotic system could attain elaborate seromuscular dissection through careful blunt dissection and accurate monopolar bleeding control even 1 cm above the peritoneal refection (Fig. 3), while retaining as many vessels as possible. Abundant blood supply and elaborate anatomy were benefit for decreased pelvic exudate and effusion which may reduce the risk of anastomotic leakage. However, due to the relatively large diameter of Trocar (8 mm) and un-securely sutured peritoneum, one case of incisional hernia occurred in the RAS group. This patient underwent a second surgery, and fortunately, necrosis did not happen in the incarcerated intestine. This alerted us to the importance of ensuring more secure sutures for the peritoneum and muscles when closing robotic surgical incisions. We also found that the initial onset of postoperative enterocolitis in patients younger than 3 months occurred significantly earlier. This finding was also consistent with a prior study [21]. The immature immunity and poor tolerance to infection of young infants, as well as the surgical stress, may be the cause of short-term complications [22, 23].

## Conclusion

In conclusion, RAS is safe and effective in treating HSCR and can result in better intra-operative and postoperative outcomes than LAS. Our findings suggest RAS is an ideal alternative in HSCR children without considering its cost and that delaying primary surgery until later in infancy (> 3 months) may improve outcomes.

## Data Availability

All data generated or analysed during this study are included in this published article.

## Abbreviations

RAS: Robotic-assisted surgery
LAS: Laparoscopic-assisted surgery
HSCR: Hirschsprung’s disease
LS: Laparotomy surgery
